# Linear and Nonlinear Regression Analysis for the Adsorption of Remazol Dye by Romanian Brewery Waste By-Product, *Saccharomyces cerevisiae*

**DOI:** 10.3390/ijms231911827

**Published:** 2022-10-05

**Authors:** Szende Tonk, Eszter Rápó

**Affiliations:** Environmental Science Department, Sapientia Hungarian University of Transylvania, Calea Turzii no. 4, 400193 Cluj-Napoca, Romania

**Keywords:** adsorption, brewery yeast, *Saccharomyces cerevisiae*, remazol brilliant red F3B dye, wastewater

## Abstract

Earth’s water balance and economy are becoming increasingly fragile due to overpopulation, global warming, severe environmental pollution and both surface and groundwater pollution. Therefore, it is essential to find solutions to the problems of water scarcity and water pollution. In this research, an experiment was designed to optimize the technique for the adsorption of Remazol Red F3B (RR) dye by lyophilized brewery yeast waste from the fermentation process. Moreover, we proved that brewery yeast is a great adsorbent. Batch adsorption experiments were carried out for optimization of different initial parameters, such as initial dye concentration (5–1000 mg/L), amount of yeast (0.5–2.5 g), pH (3–11) and temperature (20 to 40 °C). Furthermore, the structure and elemental composition of the adsorbent were analyzed with SEM, EDS and FTIR before and after biosorption. The best fits for the mathematical isotherm models in the case of the linear form were the Langmuir I and Freundlich models (R^2^ = 0.923 and R^2^ = 0.921) and, for the nonlinear form, the Khan model (R^2^ = 0.9996) was the best fit. The pseudo-second-order kinetic model showed the best fit for both linear (plotting t/q_t_ vs. t) and nonlinear forms, are the calculated q_e_ values were similar to the experimental data.

## 1. Introduction

Without access to sufficient quantities of safe drinking water of acceptable quality for the world’s population, there can be no global environmental sustainability [1]. According to recent publications, global water scarcity is increasing, thereby affecting many household consumers and industrial facilities. Furthermore, scarce freshwater resources have been further depleted as a result of climate change (major droughts, droughts, floods, extreme weather) and population growth (increased demand for quality of life products, food shortages, industrialization, disease, overuse and mismanagement) [2,3,4].

The main source of dye-containing effluents is the paper and pulp industry, as along with the by-products of leather production, but the production of dyes and the manufacture of cosmetics and pharmaceuticals also generate large quantities of effluents containing high concentrations of dyes [5]. The fashion industry relies on water throughout the production process for textiles and clothing. The amount of water used by the fashion industry has been shown to be equivalent to the annual consumption of 110 million people. The production of cotton or other textiles and their dyeing are also water-intensive, with up to 2500 L of water needed to produce just one cotton T-shirt [6]. In addition to such high water consumption, the textile industry is estimated to use the largest amount of dyes worldwide at around 10,000 tons/year, resulting in around 100 tons of dye solution wastewater. This is particularly dangerous for aquatic ecosystems and human health [7].

There are currently different techniques used for removing synthetic textile dyes from water. These include physical, chemical and biological treatment processes: filtration, sedimentation, coagulation/flocculation, biological treatment, extraction, membrane separation, ion exchange, photocatalytic degradation, oxidation and adsorption. Each of these methods has both advantages and disadvantages, which have been discussed in several previous studies. Among the conventional methods, adsorption has several advantages: it does not require excessive use of chemicals, it is fast and it is cost-effective [8,9,10,11,12].

Beer is the third most consumed drink after water and tea, making it the most popular alcoholic drink. Beer production is spread worldwide, and beer is the most popular alcoholic beverage in many countries [13]. As a result, the beer industry is of major economic importance. It has a worldwide annual revenue of USD 294.5 billion [14,15]. With up to 75 L of clean water required to make a pint of beer and with an annual production of 1.91 billion hectoliters, the beer industry is extremely dependent on one of the most endangered resources on the planet [16,17]. Its by-product is brewery yeast, which is generated during the fermentation processes and results in large quantities of waste [18,19].

In this research, the impact of the bioremediation process is analyzed with respect to two water-intensive industries, the garment and textile industry and the Romanian beverage industry (brewing), especially in view of their by-products. Despite recent research demonstrating the dye-removal potential for waste brewery yeast, biosorption studies on textile dyes (Remazol Red F3B) made from untreated waste brewery yeast (extracted in industrial form after the fermentation cycle) are not satisfactory. Even if *Saccharomyces cerevisiae* yeast is a promising (hence widely studied) model organism, the mechanism underlying the interaction between dyes and yeast is not yet fully understood. The different influencing parameters can affect the mechanism and the dye removal capacity, which makes the system vulnerable in large-scale applications. These studies are, therefore, only at the laboratory-scale phase.

Our objective is thus essential: to improve, optimize and understand the laboratory-scale biosorption of a model dye by yeast. The main focus of our research is the adsorptive capacity of lyophilized (post-fermentation) brewery yeast for an important reactive textile azo dye, Remazol Red F3B (RR). For laboratory experimental optimization, the batch adsorption method was used, and the effect of initial dye concentration, adsorbent dosage, solution pH and temperature were studied. The composition, structure and morphology of brewery yeast was analyzed using energy diffusion spectroscopy (EDS), scanning electron microscopy (SEM) and Fourier-transform infrared spectroscopy (FTIR) measurements. With the help of mathematical isotherm, kinetic, diffusion and thermodynamic models, the nature of the adsorption was investigated. For the first time, linear and nonlinear fitting of equilibrium adsorption isotherm and kinetic models of RR dye and lyophilized brewery yeast was performed.

## 2. Results and Discussion

### 2.1. Optimization through Experiments

**Figure 1:**
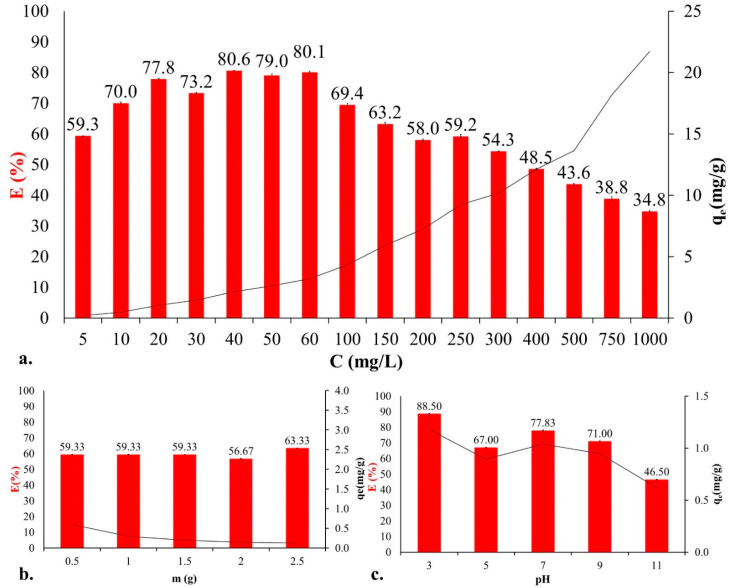
Influencing factors of adsorption: effect of (**a**) initial concentration (C_i_ = 5–1000 mg/L, 1.5 g yeast, 700 rpm, pH = 6.0 ± 0.2, T = 20 ± 1 °C), (**b**) adsorbent dosage (C_i_ = 5 mg/L, 700 rpm, pH = 6.0 ± 0.2, T = 20 ± 1 °C), and (**c**) pH (C_i_ = 20 mg/L, 1.5 g yeast, 700 rpm, T = 20 ± 1 °C), with standard deviations calculated from nine experiments and their measurements.

Several studies [20,21] have discussed the influence of the initial concentration of pollutants. Since the number of binding sites on the surface of an adsorbent is given, the concentration affects the rate of contaminant removal and the efficiency (E%) of binding H. In our previous review [9], we studied trends in the effects of initial concentration based on 19 studies. Three trends were observed based on the literature review: (i) when the C_i_ increases, the E decreases; (ii) when the C_i_ increases, the E increases; and (iii) the C_i_ does not affect the removal efficiency. In the present study, we investigated the effect of concentration over a broader range (Figure 1a). Sixteen different initial concentration values were used in the experiments, ranging from 5 to 1000 mg/L. We observed an initial increase in dye removal efficiency that reached a maximum of 80.6% at a concentration of 40 mg/L. The increase in efficiency can be explained by the fact that, at lower concentrations, more active binding sites were available for the binding of dye molecules [9]. At concentrations of 40, 50 and 60 mg/L, E was almost constant, as the active sites were saturated. After saturation, the number of binding sites was limited. The quantities in equilibrium (q_e_) were also studied. The q_e_ increased with increasing concentration (q_e;5 mg/L_ = 0.2 mg/g; q_e;1000 mg/L_ = 21.7 mg/g).

The amount of adsorbent (m; the yeast) is an important parameter because it affects the number of binding sites available. The efficiency of dye removal depends on the interaction between the adsorbent and the pollutant. Preliminary research [9,22] has shown that, in most cases, as m (g) increases, E (%) also increases. In contrast, q_e_ is negatively correlated with the change in mass. In our study, the highest efficiency was obtained with the addition of 2.5 g yeast in a 5 mg/L solution suspension (Figure 1b). On the other hand, q_e_ decreased from 0.59 mg/g to 0.13 mg/g as the mass increased.

The pH of an aqueous solution influences the adsorbent and adsorbate properties and the adsorption process. In this study, we examined the effect of pH on the adsorption of RR dye by brewery yeast (Figure 1c). RR dye, as a reactive dye, is anionic in nature; therefore, we expected it to adsorb better in an acidic medium. Our assumptions were proven correct, as the highest E (88.5%) was reached at pH = 3 and the smallest at pH = 11, where E = 46.5%.

**Figure 2:**
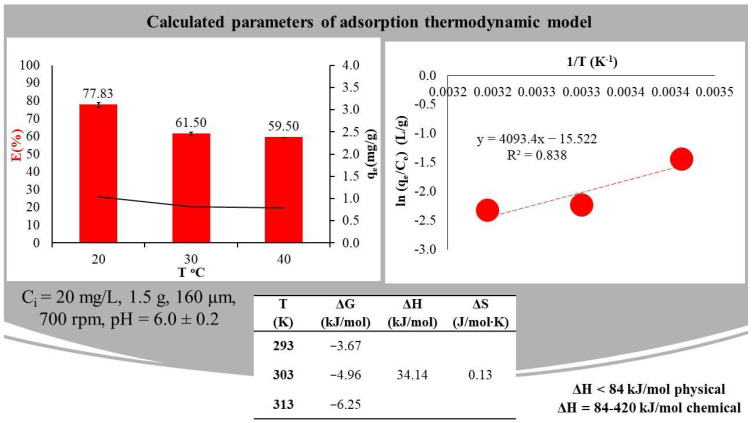
The effect of temperature on adsorption and thermodynamic parameters, with standard deviations calculated from nine experiments and their measurements.

Another physico-chemical parameter that affects the determination of the optimal conditions for adsorption is temperature. In our study, both the efficiency and q decreased with increasing temperature. At 20 °C, the efficiency/quantity in equilibrium was 94.4%/1.04 mg/g, while at 30 °C, it was 92.4%/0.82 mg/g and, at 40 °C, it was 89.7%/0.79 mg/g (Figure 2). The results indicate that the sorption can be attributed to weak van der Waals and dipole forces and bonds. As the temperature increases, the thermal motion of the molecules increases, so the adsorption decreases.

The results obtained were also used to investigate the thermodynamic parameters, which were determined for the following experimental parameters: C_i_ = 5 mg/L RR dye solution, 1.5 g yeast, 700 rpm stirring speed and pH = 6.0 ± 0.2. The calculated entropy, enthalpy and Gibbs free energy values are shown in Figure 2. It can be observed that the Gibbs free energy (ΔG) value decreased with increasing temperature (−3.67, −4.96, −6.25 kJ/mol), indicating that the spontaneity of the process was inversely proportional to temperature. The positive value for the ΔH enthalpy (34.14 kJ/mol) indicates the endothermic nature of the adsorption process. According to the literature, if ΔH < 84 kJ/mol, physical adsorption takes place, but if ΔH ranges from 84–420 kJ/mol, chemical adsorption takes place. In our experiment, the enthalpy value was around 25 kJ/mol, as a result of which the adsorption was physical. A positive value of ΔS entropy (0.13 J/molK) indicates an increase in the randomness of the adsorption process at the solid/liquid interface [23]. This indicates a heterogeneous adsorbent surface [24].

In summary, the Gibbs’ free energy values demonstrate that the biosorption of RR dye and yeast is a thermodynamically possible and spontaneous process. Based on the enthalpy value obtained, the adsorption is an endothermic process and physical adsorption takes place between the yeast and the dye. The results obtained are also very promising economically, since the removal of RR does not require energy input from heating [23,25,26,27,28].

The optimal parameters for RR dye removal with brewery yeast were C_i_ = 5 mg/L, 1.5 g biomass, 700 rpm, pH = 3 ± 0.2 and T = 20 ± 1 °C, where E = 88.5% and standard deviations calculated from nine measurements.

Following such optimizations processes, the regeneration of the adsorbent could be a further task [9,29]. Several articles discuss this matter, as it can have important economic benefits [30,31]. Therefore, in future studies, the regeneration and then the recycling of the yeast could be the main focus.

### 2.2. Characterization of Adsorbent

**Figure 3:**
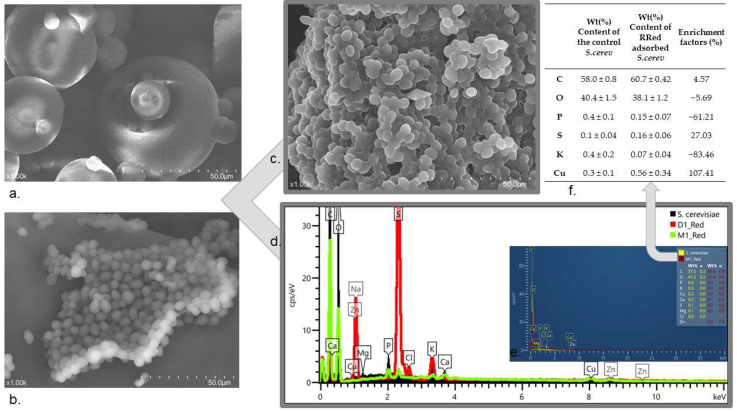
Adsorbent characterization, with SEM micrographs for (**a**) RR dye, (**b**) control *S. cerev.*, (**c**) 1 g/L RR adsorbed yeast. EDS spectra (**d**) comparison of *S. cerev.* (black), RR dye (red) and yeast sample after RR adsorption (green). (**e**) Comparison of *S. cerev.* (yellow) and yeast sample after RR adsorption (red) with numerical values. (**f**) Enrichment factor calculations based on six EDS measurements.

The surface morphological properties of the yeast and RR dye used during adsorption were studied by scanning electron microscopy (SEM). Both the control and the dye-adsorbed yeast sample were recorded (Figure 3a,c). The spherical, special-shaped cellular structure of the paint could be observed (Figure 3b). The spindle and pointed egg-shaped yeast, which are also reported in the literature, are also shown in the figure (Figure 3a). After adsorption, this porous structure disappeared, as seen in the composition of the peaks; the cells seemed to have fused and a cellular morphology difference could be seen (Figure 3c). Presumably, the structure changed because of the process and the “gaps” were saturated with the dye molecules.

The chemical composition of yeast, RR dye and the yeast sample after RR adsorption was determined with energy diffusion spectroscopy (EDS). Comparative spectra can be seen in Figure 3d,e. According to the spectra, the C, O, P, S, K and Cu elementary peaks of dye-adsorbed yeast showed different intensities compared to the control sample. The results show that the dye contained S (0.16 ± 0.06) and Cu (0.56 ± 0.34 wt%) in traces; moreover, after adsorption due to dye uptake, the yeast sample contained Cu. Based on the result of the enrichment factors (Figure 3f), the amounts of C and S increased in the sample.

**Figure 4:**
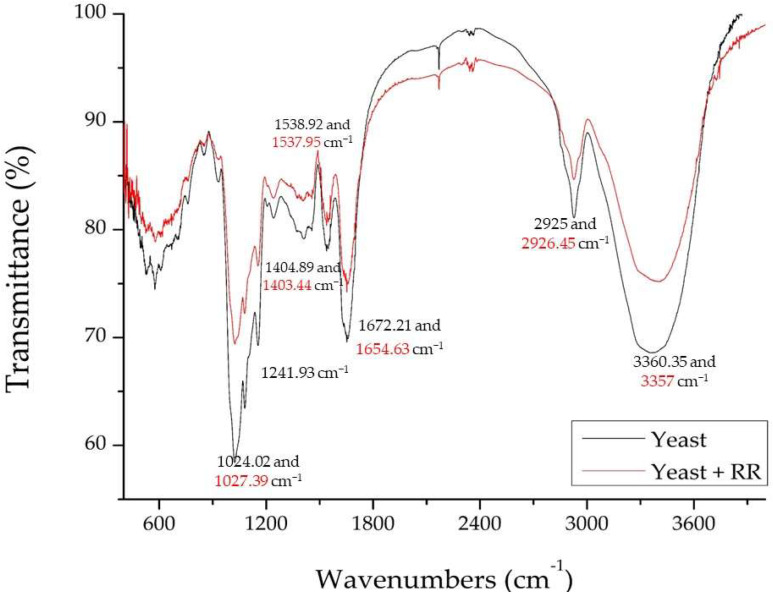
FTIR analysis for lyophilized yeast before and after RR dye adsorption.

To study the mechanism of any adsorption process, Fourier-transform infrared spectroscopy (FTIR) analysis is an essential task. The adsorption mechanism of the RR dye and lyophilized yeast also requires the identification of the functional groups involved in the dye biosorption process. Therefore, we recorded FTIR spectra in the range of wavenumbers from 400 to 4000 cm^−1^ (Figure 4).

In the characteristic infrared bands exhibited by yeast and the adsorbed dye, strong vibrations between 3000–3500 cm^−1^ indicated the presence of O-H and/or N-H functional groups. Peaks at 3360.35 and 3357 cm^−1^ (of yeast and dye-adsorbed yeast, respectively) showed the presence of amino groups (N-H of protein) and hydroxyl vibrations of carbohydrates [32].

The peaks between 3050 and 2800 cm^−1^ in the very complex spectra (2925, in our case, and 2926.45 cm^−1^ after adsorption) corresponded to the symmetric and asymmetric stretching of methyl and methylene groups in the yeast cell membrane phospholipids [33,34].

Due to the peptide bonds of the yeast cell, the amide I and amide II bands were visible in the spectrum between 1700 and 1500 cm^−1^ (presence of -C=O and N-H functional groups at 1672.21 and 1654.63 cm^−1^, respectively). Peaks were observed in yeast at 1538.92 cm^−1^ and in dye-adsorbed yeast at 1537.95 cm^−1^, corresponding to C=C stretching of aromatic rings [32,35].

As in all cells, yeast contains proteins and fatty acids; these are typically found between the wavenumbers of 1500 and 1300 cm^−1^. The spectra obtained before and after adsorption show vibrations at 1404.89 and 1403.44 cm^−1^, indicating the presence of sulfur (-SO-) and phosphorus (PO−) groups. This may also have indicated the bending of C-H or stretching of C-C [33,36,37].

The 1250 and 1000 cm^−1^ spectral region was characterized by peaks for beta-glucans, nucleic acid phosphodiester groups and phospholipids. The wavenumber 1241.93 cm^−1^ was the coupling of the stretching band of C-N and the bending band of N-H, both from amide III [38], The -C=O bond indicated chitin, one of the most important components of the yeast cell wall [24,39].

The bands observed at 1024.02 and 1027.39 cm^−1^ were assigned to the -C-O stretching vibrations of alcohols and carboxylic acids, which are mainly coupled to the complex vibrations of carbohydrates and are present in cell wall sugars. The C-C and C-N bands of the aliphatic amine functional groups confirmed their presence in the cell [34].

### 2.3. Interpretation of Adsorption Isotherm, Kinetic and Diffusion Models

The determination of the most appropriate adsorption equilibrium correlation, isotherm and kinetic models is essential to understanding new biosorbents and to achieving the ideal adsorption system. The study of these mathematical models is crucial for reliable prediction of adsorption parameters and constants. Furthermore, these empirical models provide information on quantitative comparisons of adsorbent behavior when comparison with other studies, adsorbent systems or research and experimental conditions is desired. Equilibrium relationships, commonly referred to as adsorption isotherms, describe in perspective how pollutants interact with adsorbents. They are critical for optimizing adsorption mechanism pathways. Models must be studied to express the surface properties and capacities of adsorbents and to design adsorption systems efficiently [40,41,42]. Knowledge of the adsorption equilibrium is one of the most important pieces of information because it facilitates knowledge and understanding of the adsorption process. The adsorption isotherms can be used to identify the type of adsorption (physical or chemical nature) and the surface properties of the adsorbent (homogeneous or heterogeneous surface) [43,44,45]. The adsorption capacity can be calculated and compared with results obtained experimentally. Finally, an equilibrium relationship between the adsorbent (lyophilized yeast) and the adsorbate (RR dye) can be established and identified. Consequently, investigation of the isotherms and kinetics is a critical part of adsorption research, as their understanding and interpretation can provide insight into the adsorption mechanism, thus ensuring the optimization and efficient design of the system.

Recently, one of the most widely used methods has been linear regression analysis, which reveals the best-fitting models. The linear regression method helps in quantifying the distribution of adsorbed substances, analyzing the adsorption system and checking the consistency of the theoretical assumptions about the adsorption isotherm model [46]. To calculate or predict the parameters of the models, the equations for each model were converted to linear form using the linear least squares method.

The results for the linearized isotherm models are listed in Table 1, where the linear regression coefficients (R^2^) of these models are compared and the models’ specific parameters are determined. The results were used to rank the best-fitting models in the following order: Langmuir I (R^2^ = 0.923) > Freundlich (R^2^ = 0.921) > Temkin (R^2^ = 0.898) > Langmuir II (R^2^ = 0.892) > Dubinin–Radukevich (R^2^ = 0.712) > Langmuir III (R^2^ = 0.571) > Langmuir IV (R^2^ = 0.508).

It was observed that, under our experimental conditions, the Langmuir isotherm fit the equilibrium data with the highest accuracy among the linear models. However, there was only a small difference compared to the Freundlich model. Thus, it is not clear whether adsorption occurred on a homogeneous or heterogeneous surface. The Langmuir model assumes that the adsorption is monolayer, whereas the Freundlich model suggests that the active sites and the energy are exponentially distributed. For the Langmuir I model, the separation parameter R_L_ was calculated, and the value of R_L_ indicates the type and favorability of the isotherms; i.e., irreversible if R_L_ = 0; favorable, when 0 < R_L_ < 1; linear, when R_L_ = 0; or unfavorable if R_L_ > 1. We found that R_L_ ranged from 0.13 to 0.97 (determined for 16 initial concentrations in the range 5 to 1000 mg/L), indicating favorable adsorption [47,48]. At high concentrations, the R_L_ values were close to the lower acceptable range, indicating a high degree of irreversibility. At higher concentrations, we obtained R_L_ values close to 1 (R_L-5 mg/L_ = 0.97, R_L-10 mg/L_ = 0.94).

Since the Temkin constant B (B = 4 × 10^−5^ J/mol) was less than 20 kJ/mol and the energy E (E = 4 × 10^−6^ kJ/mol) was less than 8 kJ/mol, we assumed that adsorption occurred by physisorption, forming weak van der Waals bonds between the RR dye and active sites on the surface of the adsorbent with equivalent binding sites.

From the linearized isotherm models, we could see that our results were inconclusive. Several studies report that the linearized form has significant limitations, can negatively affect the results and can introduce error potentials, thus distorting the design of an efficient adsorption system [49]. To avoid this and to ensure the comparability of the data, error analysis and optimization techniques were used [50]. With the development of computer programs, it has become possible to fit the curves of models in order to obtain experimental data with the highest possible accuracy. In this study, the results of equilibrium data with T = 20 ± 1 °C, C_i_ = 5–1000 mg/L, and m_yeast_ = 1.5 g experimental conditions were examined using nonlinear curve fitting with four two-parameter (Langmuir, Freundlich, Temkin and Dubinin–Radushkevich) and six three-parameter (Liu, Toth, Kahn, Sips, Redlich–Peterson and Radke–Prausnitz) isotherm models.

**Figure 5:**
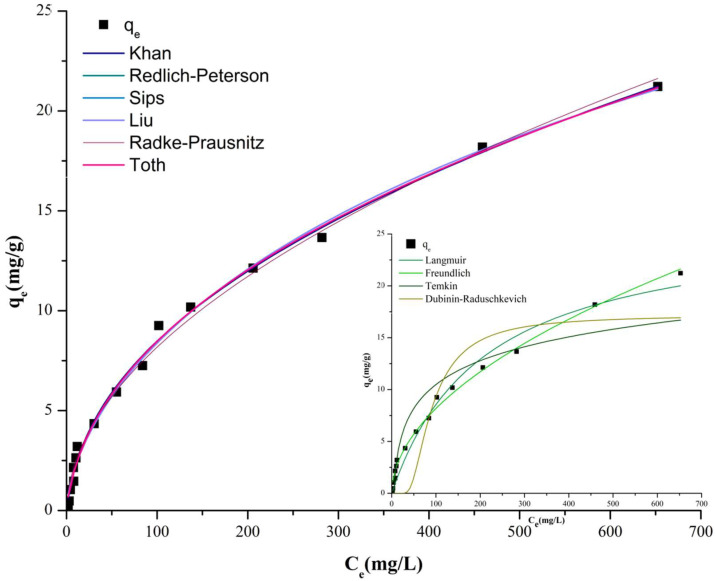
Nonlinear isotherm model fittings for RR dye adsorption onto brewery yeast surface (C_i_ = 5–1000 mg/L, 1.5 g biomass, 700 rpm, pH = 6.0 ± 0.2, T = 20 ± 2 °C).

The selection criteria for the best-fitting isotherm model necessarily include high R^2^ and low error values to compare the adequacy of each model with 95% confidence intervals. Among the isothermal models, the best results were obtained for the Khan isothermal equation, with the highest R^2^ (0.996) and low χ^2^ (3.74) and RMSE (0.49) error values (Table 2). The best-fitting models after nonlinear curve fitting were also ranked based on linear regression coefficient values (Figure 5): Khan (R^2^ = 0.9996) > Redlich–Peterson (R^2^ = 0.9957) Sips = Liu (R^2^ = 0.9948) > Radke–Prausnitz = Freundlich (R^2^ = 0.9937) > Langmuir (R^2^ = 0.9773) > Toth (R^2^ = 0.0.9251) > Temkin (R^2^ = 0.8980) > Dubinin–Radushkevich (R^2^ = 0.8465). The Kahn, Redlich–Peterson, Sips and Liu isotherms are all empirical models with three parameters. All the equations are combinations of Langmuir and Freundlich models [40,51,52]. They thus unify elements of the two most studied two-parameter models, the Langmuir and Freundlich models. Their application is proposed to reflect the joint properties of the two models mentioned above [50,53]. The models can predict the binding on a heterogeneous surface. The models are also characterized by a reduction to the Freundlich model at low adsorbent concentrations and to the Langmuir model at high concentrations. The Langmuir model then assumes single-layer adsorption [54].

Examining the two-parameter models, the Freundlich isotherm model showed better compliance compared to the conventional linear method, with a high R^2^ value (0.9937) and relatively lower error value χ^2^ (6.0) and RMSE (0.54). The Freundlich model assumed multilayer adsorption of RR dye on the surface of heterogeneous brewery yeast. The favorable nature of the adsorption was confirmed by the fact that the Freundlich constant (n_F_ = 1.904) was greater than 1 [10].

The pseudo-first-order (Lagergren) and pseudo-second-order (Ho and McKay) kinetic models with linear and nonlinear fits were used to study the adsorption kinetics of RR dye and brewery yeast. These models can help find a relationship between time and the adsorption process, since they allow calculation of the rate of dye removal and, thus, the contact time required for remediation. In our study, we investigated the rate of dye removal and, simultaneously, the amount of adsorbed material between 1 and 360 min until equilibrium values were obtained [55]. Table 3 shows the types of kinetic models, calculated parameters, regression coefficients and, for nonlinear fits, the standard deviations of the parameters, the reduced χ^2^ and the adjusted R^2^.

It can be observed that:

- The pseudo-first-order model was not sufficiently accurate in the analysis of kinetic data for either linear or nonlinear fits;
- The R^2^ values of the pseudo-first-order model were low, and the calculated q_e_calc_ differed greatly from the experimental results;
- Among the linearized pseudo-second-order kinetic models, pseudo II.1 showed more accurate results, where t/q_t_ was plotted as a function of time t in the linearization (q_e_ = 1/slope and k_2_ = slope^2^/intercept can be calculated from the equation linearized equation);
- The pseudo-second-order kinetic model, both the linearized (pseudo II.1) and the nonlinear models, applied with high accuracy for all the different concentrations;
- The pseudo-second-order kinetic model was a better representation of the kinetic behavior and, thus, more suitable for the determination of the rate constant and q_e_calc_;
- Excellent R^2^ values and good correlation between experimental (q_e_exp_) and calculated (q_e_calc_) values were obtained (pseudo II.1);
- The initial sorption rate h (g/mg/min; h = k_2_ × q_e_^2^) increased with the increase in initial dye concentration (h = 0.18, 0.72, 1.64, 3.02, 2.58, 3.98, 5.66), indicating the presence of a strong driving force for the mass transfer and an increased number of available active sites [56].

Overall, the experimental data showed a good fit to the pseudo-second-order kinetic equation, and the correlation coefficients (R^2^) of the linear plots were greater than 0.993 for all experimental data (Table 2, linear–pseudo II.1). In this case, the calculated q_e_ values were also consistent with the experimental data. The adsorption system thus followed the pseudo-second-order kinetic model. However, the model assumed that the rate was determined by chemical adsorption.

Adsorption, being a mass transfer process, involves three main steps: (i) an external (liquid-film) diffusion, where the dye molecules transfer through a liquid film to the yeast adsorbent; (ii) internal (intra-particle) diffusion, where diffusion occurs in the pores of the yeast; and (iii) actual adsorption on the surface of the yeast [57]. Based on this process, the kinetic models can be divided into two main groups [58]:

- Those describing the relationship between contaminant molecules or ions (RR dye) and active centers or binding sites on the surface of the adsorbent (brewer yeast), including pseudo-first-order and pseudo-second-order kinetic models;
- Diffusion models, which assume that in actual water treatment there is immediate diffusion between the contaminant and the active sites.

Therefore, the study of diffusion is an important step in understanding the mechanism of the dye removal process. When intra-particle and film diffusion were plotted, the intercept points did not pass through the origin (intercept_id_: 0.122–2.501 and intercept_fd_: 1.05–1.49); this led to the conclusion that the two diffusion models were not the only rate-determining factors. Table 4 shows the diffusion coefficient values D, which were in the range from 10^−5^ to 10^−13^ cm^2^/s.

According to Yakout, if the values of the diffusion coefficients D are in the above mentioned interval, then intra-particle diffusion is a rate-limiting factor when chemical bonding takes place [58]. For better understanding, the initial adsorption factor R_i_ and the initial point of the kinetic curve C/q_ref_ were calculated (Table 5). As R_i_ was smaller than 0.1 (−9 and −2.6) and C/q_ref_ was higher than 0.9 (10 and 3.6), the initial adsorption behavior was set in zone 4, indicating an approach to complete initial adsorption [59]. Adsorption occurred right at the beginning of the water-cleaning process. According to [59], this phenomenon is not usual in adsorption processes (it is typical of aggregation and coagulation) but can occur in cases of powdered adsorbents.

Overall, the obtained results (diffusion coefficient values D ranging between 8.31 × 10^−9^ and 3.34 × 10^−8^ cm^2^/s), as well as the fact that the intercept points did not pass through the origin, led to the conclusion that the two diffusion models were not the only rate-determining factors, and the biosorption process affected adsorption speed.

The results of each model indicated both physical and chemical mono- or multilayer adsorption on heterogeneous surfaces; therefore, many mechanisms (physical (van der Waals) and chemical (π–π) interactions) may be involved in the adsorption of RR dye and brewery yeast.

### 2.4. Possible Adsorption Mechanism

In our research, we used yeast (lyophilized) production residues from a brewery in Romania to remove organic dye from water. The yeast we worked with mostly contained dead yeast cells leftover after a few fermentation cycles during brewing and is a waste product of the industry. This industrial by-product was lyophilized and used without any yeast treatment. We had no knowledge of the percentage of lyophilized yeast cells that were alive or dead. Our research—and, therefore, our modeling parameters and mechanisms—were based on the assumption that the cells were dead.

All in all, after the fermentation cycle, the available biomass could have contained both living and dead cells. Live yeast cells can perform stain removal in two ways. The dye may be adsorbed on the cell wall of the yeast, penetrate the cell wall and accumulate inside the cell or biodegradation can occur through various enzymes (oxidases and reductases) [60]. Irrespective of the nature of the yeast cell (living or dead), biosorption occurs between the contaminant (RR dye) and the yeast cell wall, may be due to electrostatic interaction, complexation, chelation and microprecipitation, ion exchange or physical and chemical adsorption, depending on a number of factors [61]. The mechanism of dye biosorption on yeast is a sophisticated and multi-faceted process that is not fully understood, and it may involve more than one mechanism [62].

The biosorption capacity of yeast cells and the mechanism of dye removal can be influenced by the nature of the biomass (living or dead yeast cell), the functional groups on the cell wall, the number of reactive binding sites and their availability and the affinity (i.e., binding strength) between the sites and the dye. The nature of the dye molecule (anionic or cationic) and the physico-chemical conditions of the adsorption treatment (contact time, impurity concentration, amount of yeast, pH, temperature) may also play a role [63].

**Figure 6:**
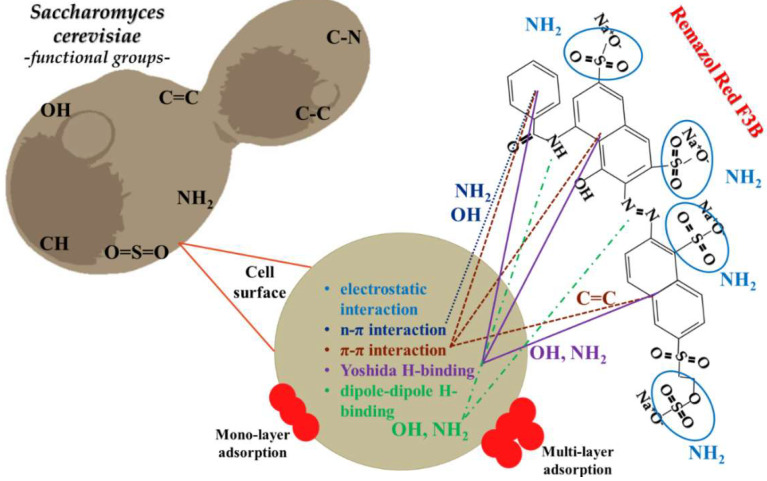
Proposed interaction of RR dye with *Saccharomyces cerevisiae* brewery yeast.

Early biosorption studies from recent years generally used living cells, but it has been found that dead yeast cells may have the same or even higher binding capacity [57]. Some studies have described the structure and components of the *S. cerevisiae* yeast cell and the functional groups that occur on its surface. Its composition includes proteins, amino acids, polysaccharides and lipids. Accordingly, carboxyl, hydroxyl, amide, amino, phosphate and other charged groups have been identified, demonstrating strong binding forces with the dye molecules. It has been shown that the biosorption process can also be achieved by chelation and the formation of ionic bridges between the dye molecules and the functional groups [61] Figure 6 represents the proposed mechanisms.

In the present paper, changes in the structural and elemental composition of lyophilized yeast cells and Remazol Red dye adsorbed on the yeast were investigated by SEM, EDX and FTIR analysis. We found that the structure underwent morphological changes as a result of adsorption fixation and that the sulfur and copper typical of the dye were detectable in the samples.

FTIR studies revealed the presence of several functional groups. Accordingly, the adsorption of the dye on the yeast cell wall was assumed to be mediated by three main sources:

- Amide and amine bonds (R-NH-C-O-CH_3_-C-NH, −C-NH);
- (−C=O) and (−C-O) bonds, which are part of the chitin structure found in the sugar in the cell wall;
- The -C-N-C group found in the cell wall protein of yeast [24].

Depending on the type of interaction between the adsorbent surface and the contaminant, the biosorption process can be divided into two types: (i) chemical adsorption, an irreversible process resulting in the formation of strong chemical bonds; and (ii) physical adsorption, which is reversible and, in most cases, characterized by weak van der Waals forces, H-bonds, polarity and dipole–dipole H-bonding interactions. Furthermore, FTIR studies have demonstrated that Yoshida H-bonding, dipole–dipole H-bonding and π–π and n–π interactions can occur upon adsorption of dye molecules on yeast cells (Figure 6) [32,35,61].

## 3. Materials and Methods

### 3.1. Adsorbent and Adsorbate

*Saccharomyces cerevisiae* yeast (BY) was supplied by a brewery factory in Romania, Miercurea Ciuc and was used as a biosorbent after preparation. In the brewing process, after a few fermentation cycles, the brewery yeast by-product was lyophilized. For lyophilization of yeast in aqueous solution, we used the Telstar Cryodos 50 lyophilization system, which operates at −30 °C and 4 × 10–2 mbar pressure. The yeast solution was placed in 50 mL centrifuge tubes and frozen at −80 °C, and then mounted on the distribution tubes of the lyophilization system. The lyophilization was carried out until the samples were completely dry (24 h).

**Figure 7:**
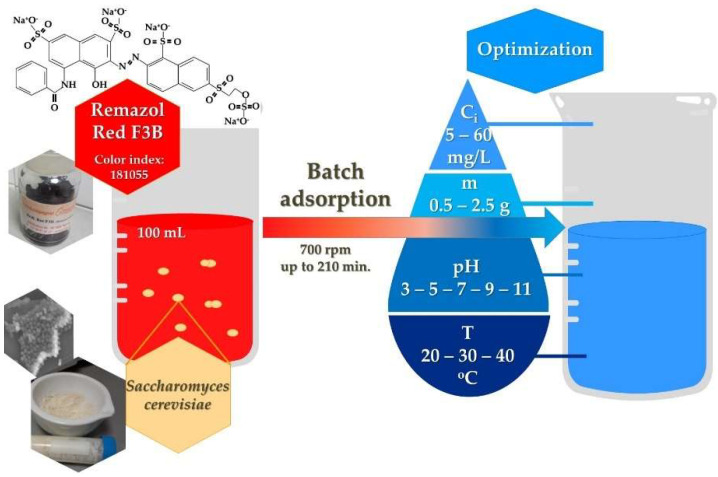
Graphical representation of the adsorption experiment with optimization initial parameters.

Remazol Brilliant Red F3B (RR; C_29_H_19_N_3_Na_4_O_17_S_5_; MW: 933.76 g/mol) dye of analytical grade was purchased from DyeStar Singapore Pte. Ltd. and was further used throughout the experiments without any purification (Figure 7).

### 3.2. Adsorption Optimization

The batch adsorption method was used to remove RR dye from aqueous solution (100 mL) with BY (in a 250 mL Erlenmeyer flask) until adsorption equilibrium was reached. A stock solution of 2 g/L RR dye was produced and further diluted during the experiments. The R^2^/n = 0.9997/6 calibration curve quantitative measuring technique was used for concentration determination with an Agilent Cary 60 UV-VIS spectrophotometer at λ_max_ = 539 nm.

- The efficiency of liquid-phase adsorption, the sorption performance, is influenced by a number of physicochemical factors. To determine the optimum conditions, experiments were carried out with different initial parameters;
- The effects of initial RR dye concentration and contact time were studied at 5–1000 mg/L concentrations. Constant experimental parameters: 1.5/100 g/mL yeast, 20 °C, 700 rpm agitation speed, pH 6;
- During the study of the 0.5/1/1.5/2/2.5 g adsorbent dosages, yeast was added to a 5 mg/L dye solution at room temperature, which was agitated at 700 rpm and pH 6;
- The pH of the dye solution was adjusted between 2 and 11 with HCl or NaOH solutions in order to study the pH. Constant parameters: 1.5 g yeast, 100 mL of 20 mg/L RR dye, stable room temperature and stirring at 700 rpm;
- With the help of a IKA C-MAG HS7 magnetic shaker, the effect of temperature (20, 30, 40 °C) on adsorption was investigated. Samples contained 100 mL 20 mg/L RR dye solution stirred constantly at 700 rpm with 1.5 g yeast.

Based on further adsorption equilibrium data analyses (efficiency E and quantity in equilibrium q_e_), mathematical isotherm, kinetic and diffusion models were calculated.

### 3.3. Analytical Studies

Several analytical techniques can be used to study the biosorption process and characterize the contaminants, the adsorbents used and the adsorption mechanism linking them. These studies were carried out in Cluj-Napoca, Romania at the National Institute for Research and Development of Isotopic and Molecular Technologies, INCDTIM.

Scanning electron microscopy (SEM) is one of the most important tools for analyzing the surface and morphology of adsorbents. The primary advantage of SEM is the high resolution that can be achieved during the examination, making it an important instrument for materials analysis. SEM provides magnified images showing the size, shape, composition and other physical and chemical properties of the sample—in this case yeast—before and after the adsorption process. The surface of the yeast before (control sample) and after (3 g of BY kept in 2 g/L dye solution for 24 h) adsorption of the RR dye powder was studied with a JEOL (USA) JSM 5510 LV SEM scanning electron microscope at various magnifications. Furthermore, these samples were further examined to determine their elemental composition with a Scanning Jeol JEM 5510 JV and Oxford Instruments EDS Analysis System Inca 300 (UK) [22].

Fourier-transform infrared spectroscopy data were obtained using a FT/IR 4100, Jasco spectrophotometer in the wavelength range from 400 to 4000 cm^−1^, and the observed bands were analyzed using ORIGIN PRO 8.5 software, OriginLab. Samples were prepared as follows: approx. 4 mg of sample was co-moistened with an agate pestle and mortar with approx. 2 g of 0.2 g KBr of this homogenized mixture was pressed under vacuum (Atlas 25T Hand Hydraulic Press, Specac) in a 13 mm diameter mold with a force equivalent to 7 t for 1 min. The spectra of the samples were recorded against a reference sample (pellet) with a content of 0.2 g KBr.

### 3.4. Isotherm, Kinetic and Diffusion Modeling

**Figure 8:**
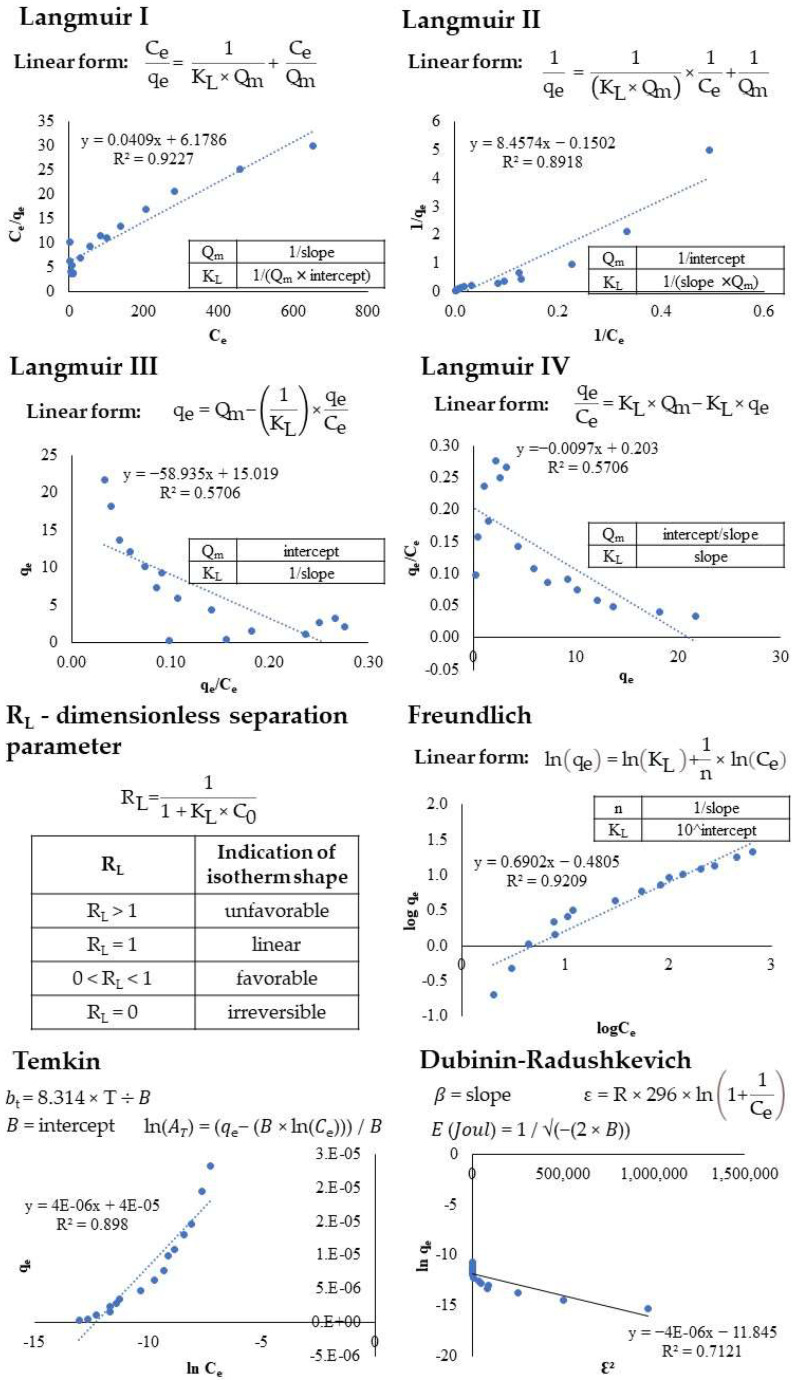
Representation of isotherm models in linear form.

In order to explore the feasibility of adsorption, four two-parameter linear isotherm models (Langmuir, Freundlich, Temkin, Dubinin–Radushkevich) were applied and are shown in Figure 8. Models were calculated for adsorption experiments where 1.5 g/100 mL BY was constantly stirred (700 rpm) in batch mode with 5–1000 mg/L RR dye for 330 min, pH = 6, T = 20 ± 1 °C.

Nonlinear isothermal and kinetic modeling could more accurately describe the function of the data. In this study, nonlinear versions of the two-parameter Langmuir, Freundlich, Temkin and Dubinin–Radushkevich and three-parameter Liu, Toth, Kahn, Sips, Redlich–Peterson and Radke–Prausnitz isotherm models were investigated with the help of OriginPro 8.5 software. The performances of these model predictions and the robustness of the results obtained were validated using statistical measures (namely, residual sum of squares error (ERRSQ/SSE), Chi-square (χ2), coefficient of determination (R^2^), average relative error (ARE), hybrid fractional error function (HYBRID), Marquardt’s percent standard deviation (MPSD) and root mean square error (RMSE, polynomial p: the number of terms)) [41,42,46].

As a next step, in cases of kinetic models, the Lagergren pseudo-first-order and the Ho and McKay pseudo-second-order (six types of linear forms) models were studied. Moreover, intra-particle and liquid film diffusions were calculated for a better understanding of the mechanism.

## 4. Conclusions

In our study, we investigated the morphology, elemental composition and adsorption capacity (RR dye removal) of brewery yeast (*Saccharomyces cerevisiae*) residue, which was left as waste after the fermentation process during brewing. The optimal laboratory conditions were determined (C_i_ = 5 mg/L, 1.5 g biomass, 700 rpm, pH = 3 ± 0.2, T = 20 ± 1 °C, where E = 88.5%).

In the characterization of the adsorbent, we observed from SEM images that the cellular, porous structure of the lyophilized yeast was fused, and morphological changes occurred. In EDX elemental analysis, the S (0.16 ± 0.06) and Cu (0.56 ± 0.34 wt%) characteristics of the dye were detected in the adsorbent after adsorption. In the FTIR analysis, the fission groups corresponding to the peaks appearing in the spectra were identified.

For the linearized isothermal models, a sequence was established based on R^2^ values: Langmuir I (R^2^ = 0.923) > Freundlich (R^2^ = 0.921) > Temkin (R^2^ = 0.898) > Langmuir II (R^2^ = 0.892) > Dubinin-Radukevich (R^2^ = 0.712) > Langmuir III (R^2^ = 0.571) > Langmuir IV (R^2^ = 0.508). The Langmuir I model proved to be the best fit. The R_L_ values confirmed favorable adsorption (from 0.13 to 0.97).

The constants of the isothermal models indicated physically good adsorption. A sequence for the nonlinearized isothermal models was also established: Khan (R^2^ = 0.99960) > Redlich–Peterson (R^2^ = 0.9957) Sips = Liu (R^2^ = 0.9948) > Radke–Prausnitz = Freundlich (R^2^ = 0.9937) > Langmuir (R^2^ = 0.9773) > Toth (R^2^ = 0.0.9251) > Temkin (R^2^ = 0.8980) > Dubinin–Radushkevich (R^2^ = 0.8465).

Seven main conclusions were drawn after linear and nonlinear fitting of the kinetic models, and in both cases the pseudo-second-order models showed higher fits. Based on data from the literature and our results, a possible mechanism for the adsorption of RR and yeast was proposed.

## Figures and Tables

**Table 1:** Calculated parameters for linearized isotherm models for RR dye adsorption onto brewery yeast surface (C_i_ = 5–1000 mg/L, 1.5 g biomass, 700 rpm, Ph = 6.0 ± 0.2, T = 20 ± 2 °C).

Langmuir I			Langmuir II			Langmuir III			Langmuir IV		
K_L_	q_max_	R^2^	K_L_	q_max_	R^2^	K_L_	q_max_	R^2^	K_L_	q_max_	R^2^
(L/mg)	(mg/g)		(L/mg)	(mg/g)		(L/mg)	(mg/g)		(L/mg)	(mg/g)	
0.01	24.45	0.923	0.02	3.66	0.892	0.02	15.02	0.571	0.01	20.93	0.508
	**Freundlich**			**Dubinin–Radushkevich**			**Temkin**				
	**n**	**K_f_**	**R^2^**	**β**	**E**	**R^2^**	**A_T_**	**B**	**R^2^**		
		**(mg^(1−1/n)^L^1/n^/g)**		**(mol^2^ kJ^2^)**	**(kJ/mol)**		**(L/g)**	**(J/mol)**			
	1.45	3.02	0.921	4 × 10^−6^	0.35	0.712	2.35	4 × 10^−5^	0.898		

**Table 2:** Calculated parameters of nonlinear isotherm models for RR dye adsorption onto brewery yeast surface (C_i_ = 5–1000 mg/L, 1.5 g biomass, 700 rpm, pH = 6.0 ± 0.2, T = 20 ± 2 °C).

Isotherm Model	Factors for the Model			Statistical Results						
				R^2^	ERRSQ/SSE	Chi-Square	ARE	RMSE	HYBRID	MPSD
**Langmuir**	**q_m_**	**K_L_**		0.9773	14.68	3.60	16.51	1.02	18.87	186.77
	0.005	27.165								
**Freundlich**	**K_F_**	**n_F_**		0.9937	4.09	6.00	−42.22	0.54	−48.25	477.66
	0.728	1.904								
**Temkin**	**b_t_**	**K_T_**		0.8980	66.03	48.06	98.96	2.17	113.09	1119.58
	3.339	0.235								
**Dubinin–Radushkevich**	**q_D-R_**	**K_D-R_**		0.8465	99.40	20.39	51.70	2.66	59.08	584.89
	17.351	0.001								
**Toth**	**q_m_**	**K_Toth_**	**n_Toth_**	0.9251	1246.32	92.26	74.04	9.44	84.62	837.72
	1.233	0.267	0.551							
**Kahn**	**q_m_**	**K_K_**	**a_K_**	0.9960	2.56	1.86	−19.61	0.43	−22.42	221.92
	2.241	0.162	0.514							
**Liu**	**q_m_**	**K_Liu_**	**n_Liu_**	0.9948	3.38	3.74	−30.67	0.49	−35.05	347.01
	86.992	0	1.660							
**Sips**	**q_m_**	**K_S_**	**n_S_**	0.9948	3.38	3.76	−30.76	0.49	−35.15	348.00
	87.317	0.007	0.602							
**Redlich–Peterson**	**K_RP_**	**a**	**n**	0.9957	2.80	2.35	−22.72	0.45	−25.96	257.02
	0.599	0.484	0.551							
**Radke–Prausnitz**	**q_m_**	**K**	**n**	0.9937	4.09	5.99	−42.18	0.54	−48.21	477.25
	0.728	1.06 × 10^46^	0.475							

**Table 3:** Calculated parameters for linear and nonlinear kinetic models for RR dye adsorption onto brewery yeast surface (C_i_ = 5–60 mg/L, 1.5 g biomass, 700 rpm, pH = 6.0 ± 0.2, T = 20 ± 2 °C).

Kinetic Model	Pseudo I			Pseudo I					
Type of Fitting	LINEAR: ln(1-q_t_/q_e_) vs. t			NONLINEAR: q_e_ × (1-exp(−k_1_ × t))					
**Parameters**	**k_1_**	**R^2^**	**q_e_calc_.**	**q_e_calc_.**		**k_1_**		**Statistics**	
				Value	Standard Error	Value	Standard Error	Red. χ^2^	Adj. R^2^
**5 mg/L**	0.008	0.902	0.06	0.17	0.01	0.62	0.17	0.001	0.286
**10 mg/L**	0.023	0.916	0.43	0.43	0.01	0.89	0.16	0.001	0.535
**20 mg/L**	0.017	0.953	0.39	0.95	0.02	0.92	0.18	0.007	0.433
**30 mg/L**	0.015	0.947	0.60	1.37	0.03	1.10	0.17	0.009	0.509
**40 mg/L**	0.011	0.973	0.36	1.98	0.05	0.69	0.12	0.036	0.461
**50 mg/L**	0.012	0.979	0.40	2.42	0.06	0.88	0.16	0.046	0.423
**60 mg/L**	0.020	0.978	0.39	2.97	0.07	1.06	0.22	0.072	0.308
**Kinetic model**	**Pseudo II.1**			**Pseudo II.2**			**Pseudo II.3**		
**Type of fitting**	**LINEAR: t/q_t_ vs. t**			**LINEAR: 1/t vs. 1/q_t_**			**LINEAR: 1/q_t_ vs. 1/t**		
**Parameter**	**k_2_**	**R^2^**	**q_e_calc_.**	**k_2_**	**R^2^**	**q_e_calc_.**	**k_2_**	**R^2^**	**q_e_calc_.**
**5 mg/L**	0.84	0.9933	0.20	10.32	0.609	0.58	5.52	0.609	1.02
**10 mg/L**	1.44	0.9997	0.47	4.48	0.895	0.51	3.92	0.895	0.57
**20 mg/L**	0.42	0.9995	1.04	2.18	0.817	0.47	1.73	0.817	0.59
**30 mg/L**	0.46	0.9999	1.46	1.75	0.884	0.41	1.53	0.884	0.46
**40 mg/L**	0.14	0.9997	2.15	0.88	0.760	0.57	0.64	0.760	0.77
**50 mg/L**	0.13	0.9996	2.64	0.83	0.797	0.49	0.64	0.797	0.62
**60 mg/L**	0.15	0.9998	3.21	0.81	0.725	0.41	0.56	0.725	0.58
**Kinetic model**	**Pseudo II.4**			**Pseudo II.5**			**Pseudo II.6**		
**Type of fitting**	**LINEAR: 1/q_t_ vs. 1/t**			**LINEAR: 1/q_t_ vs. 1/t**			**LINEAR: 1/q_t_ vs. 1/t**		
**Parameter**	**k_2_**	**R^2^**	**q_e_calc_.**	**k_2_**	**R^2^**	**q_e_calc_.**	**k_2_**	**R^2^**	**q_e_calc_.**
**5 mg/L**	0.20	0.928	0.07	2.56	0.512	0.26	0.18	0.512	0.14
**10 mg/L**	1.90	0.635	0.07	0.67	0.865	0.81	0.45	0.865	0.72
**20 mg/L**	0.41	0.871	0.43	0.27	0.771	1.92	1.00	0.771	1.51
**30 mg/L**	0.26	0.906	0.48	0.13	0.850	3.30	1.42	0.850	2.83
**40 mg/L**	0.15	0.887	0.54	0.19	0.716	3.28	2.06	0.716	2.40
**50 mg/L**	0.11	0.889	1.95	0.11	0.747	4.70	2.53	0.747	3.58
**60 mg/L**	0.90	0.551	0.04	0.06	0.693	6.94	3.11	0.693	4.92
**Kinetic model**	**Pseudo II**						**Experimental Result**		
**Type of fitting**	**NONLINEAR: q_e_^2^** **×** **k_2_** **×** **t/(1 + k_2_** **×** **q_e_** **×** **t)**								
**Parameter**	**q_e_calc_.**		**k_2_**		**Statistics**				
	Value	Standard Error	Value	Standard Error	Red. χ^2^	Adj. R^2^	**q_e_exp_.**		
**5 mg/L**	0.18	0.01	5.83	1.85	3.70 × 10^−4^	0.583	**0.20**		
**10 mg/L**	0.45	0.01	3.59	0.49	2.87 × 10^−4^	0.894	**0.47**		
**20 mg/L**	0.99	0.02	1.68	0.30	0.002	0.806	**1.04**		
**30 mg/L**	1.41	0.02	1.51	0.20	0.002	0.864	**1.46**		
**40 mg/L**	2.04	0.03	0.62	0.11	0.014	0.788	**2.15**		
**50 mg/L**	2.50	0.04	0.64	0.11	0.017	0.782	**2.63**		
**60 mg/L**	3.07	0.05	0.60	0.11	0.026	0.748	**3.20**		

**Table 4:** Calculated parameters of intra-particle and liquid-film diffusion models for RR dye adsorption onto yeast surface (C_i_ = 5–60 mg/L, 1.5 g biomass, 700 rpm, pH = 6.0 ± 0.2, T = 20 ± 2 °C).

		Intra-Particle Diffusion			Liquid-Film Diffusion		
C (mg/L)	D (cm^2^/s)	k_ip_ (mg/g∙min^1/2^)	Intercept	R^2^_ip_	k_fd_ (1/min)	Intercept	R^2^_fd_
5	8.31 × 10^−9^	0.006	0.122	0.956	0.008	1.05	0.902
10	3.34 × 10^−8^	0.011	0.355	0.752	0.023	1.53	0.916
20	2.19 × 10^−8^	0.021	0.787	0.846	0.017	1.48	0.953
30	3.34 × 10^−8^	0.021	1.191	0.767	0.015	1.82	0.947
40	1.53 × 10^−8^	0.037	1.583	0.837	0.011	1.44	0.973
50	1.68 × 10^−8^	0.043	2.001	0.865	0.012	1.49	0.979
60	2.43 × 10^−8^	0.055	2.501	0.841	0.020	1.48	0.971

**Table 5:** Calculated initial adsorption factor R_i_ and initial point of the kinetic curve C/q_ref_ for RR dye adsorption onto brewery yeast surface (C_i_ = 5–60 mg/L, 1.5 g biomass, 700 rpm, pH = 6.0 ± 0.2, T = 20 ± 2 °C), where R_i_ = 1-C/q_ref_, C = initial adsorption amount, q_ref_ = final adsorption amount.

C	q_ref_	C/q_ref_	R_i_
2.00	0.20	10.0	−9.0
3.00	0.47	6.4	−5.4
4.43	1.04	4.3	−3.3
8.03	1.46	5.5	−4.5
7.77	2.15	3.6	−2.6
10.5	2.63	4.0	−3.0
11.97	3.20	3.7	−2.7
